# Comments on Hardell and Carlberg Increasing Rates of Brain Tumors in the Swedish National Inpatient Register and the Causes of Death Register. *Int. J. Environ. Res. Public Health* 2015, *12*, 3793–3813

**DOI:** 10.3390/ijerph120911662

**Published:** 2015-09-17

**Authors:** Anders Ahlbom, Maria Feychting, Lars Holmberg, Lars Age Johansson, Tiit Mathiesen, David Pettersson, Joachim Schüz, Mats Talbäck

**Affiliations:** 1 Institute of Environmental Medicine, Karolinska Institutet, P.O. Box 210, SE-171 77 Stockholm, Sweden; E-Mails: maria.feychting@ki.se (M.F.); david.h.pettersson@ki.se (D.P.); mats.talback@ki.se (M.T.); 2 Regional Cancer Center, Uppsala/Örebro and The National Board of Health and Welfare, SE-112 59 Stockholm, Sweden; E-Mail: lars.holmberg@akademiska.se; 3 Nordic Collaborating Centre for Classifications in Health Care, P.O. Box 7000, St. Olavs Pass, NO-0130 Oslo, Norway; E-Mail: lars.age@bredband.net; 4 Department of Clinical Neuroscience, Karolinska Institutet, SE-171 77 Stockholm, Sweden; E-Mail: tiit.mathiesen@karolinska.se; 5 Neurosurgery Clinic at the Karolinska University Hospital, SE-171 76 Solna, Sweden; 6 Section of Environment and Radiation, International Agency for Research on Cancer, 150 Cours Albert Thomas, 69372 Lyon, France; E-Mail: SchuzJ@iarc.fr

Hardell and Carlberg claim in a recent article that both the Cause of Death Register and the National Inpatient Care Register indicate that there was a severe and increasing underreporting of malignant brain tumors to the Swedish Cancer Register during recent years [1]. As a consequence, they claim, the Swedish Cancer Register fails to report that malignant brain tumor incidence rates have in fact increased since 2007/2008. They suggest that this increase might be due to an increasing exposure to the population from radiofrequency electromagnetic fields emanating from mobile communications. 

**Figure 1:**
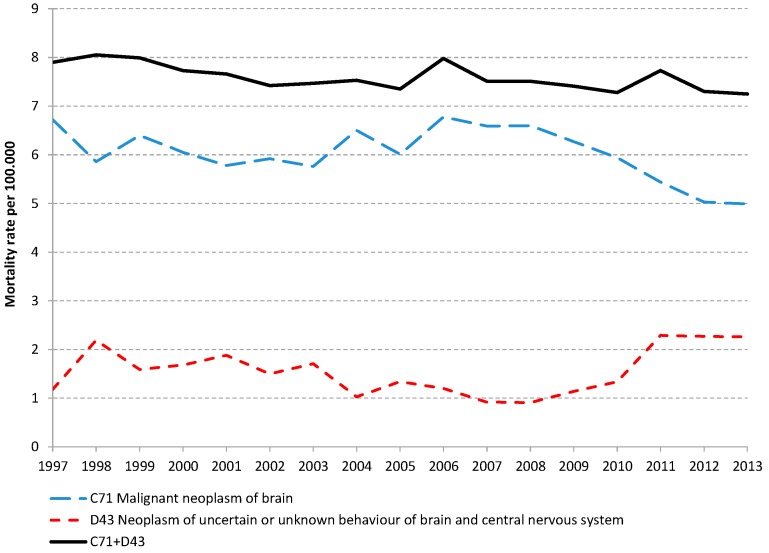
Age standardized mortality rates from the Swedish Cause of Death Register.

Their claim is based on the observation that tumors of unknown type in the brain or CNS (ICD10 D43) in both the Cause of Death Register and the National Inpatient Care Register have been increasing since 2007/2008. There are several problems with the authors’ use of these data, one of which is displayed in Figure 1. The figure shows age-standardized death rates for malignant brain tumors (ICD10 C71) in the middle line and brain and CNS tumors of unknown type in the bottom line. The top line displays the sum of the two lower lines. Two things are obvious from the figure. First, the two lower lines are closely related and one goes up when the other goes down. Second, the trend for the two diagnostic categories taken together is flat. That is, the Cause of Death Register does not indicate that malignant brain tumors have been increasing during recent years and the claim by the authors is simply not correct. The basis for their conclusion is the rise of the death rates for D43 from 2008 and onwards, at an annual rate of 22%. The real explanation to this trend is readily available in a reference from the Register [2]. The explanation is that the Register decided to speed up the registration process by making fewer requests for more detailed information by accepting the coding of more tumors as unspecified type but with no effect on the total number of brain tumors.

The results that are presented from the National Inpatient Care Register are based on hospital discharges retrieved from tables published by the National Board of Health and Welfare and not relevant for analysis of cancer incidence trends. The coverage rate of the Swedish Cancer Register has been examined by means of a comparison with data extracted from the register of hospital discharges [3]. A substantial underreporting was found for tumors of the central nervous system for ages above 70 years (44% for men and 30% for women). The underreporting for men and women below the age of 70 was however modest, 5.5 and 7.1% respectively. Upon scrutiny of medical records, it was found that over 50% of tumors that were reported to the hospital discharge registry during 1998, but were not found in the cancer register during the same year, should not have been reported to the cancer register. The main reasons were that it was not cancer or it was cancer, but the tumor should not be included as an incident case for the year evaluated. For the underreporting to hide a trend in cancer incidence it would have to get bigger with time, but no information is available about this.

Although the figures that are presented in the article may well be correct, their interpretation is grossly misleading. Already the title is misleading because the rates of brain tumor are not increasing in the Cause of Death Register. The reason for the rise of the subtype of brain and CNS tumor rates of unknown type is known and documented in the open literature. The authors actually mention themselves the close correlation between the trends of the unspecified and the malignant tumors in the Cause of Death Register, but choose to disregard it in their interpretation of data.
